# LncRNA MIR31HG is induced by tocilizumab and ameliorates rheumatoid arthritis fibroblast-like synoviocyte-mediated inflammation via miR-214-PTEN-AKT signaling pathway

**DOI:** 10.18632/aging.203644

**Published:** 2021-11-09

**Authors:** Liang Cao, Haifeng Jiang, Jing Yang, Jun Mao, Guofeng Wei, Xiangyun Meng, Hongmei Zang

**Affiliations:** 1 School of Pharmacy, Anhui Medical University, Hefei, Anhui, China; 2 Department of Pharmacy, The Second People’s Hospital of Hefei, Hefei, Anhui, China; 3 Institute of Clinical Pharmacology, Anhui Medical University, Hefei, Anhui, China; 4 Department of Emergency, The Second People’s Hospital of Hefei, Hefei, Anhui, China

**Keywords:** RA-FLS, tocilizumab, MIR31HG, miR-214, PTEN

## Abstract

Fibroblast-like synoviocytes (FLS) obtained from the joint synovium of rheumatoid arthritis (RA) patients exhibit hyperplasia and aggressive inflammatory phenotypes. This study was designed to explore the anti-inflammatory mechanism of IL-6R inhibitor, tocilizumab, in FLS-mediated inflammation in RA from the perspective of non-coding RNAs (ncRNAs). To this end, we sorted primary FLS obtained from the synovium of patients with RA and cultured them *in vitro.* The cells were then treated with tocilizumab and subjected to lncRNA- and miRNA-seq to identify the ncRNAs regulated by tocilizumab treatment using bioinformatic analysis and experimental verification. Tocilizumab treatment enhanced the expression of lncRNA MIR31HG and reduced that of micoRNA-214 (miR-214). In addition, miR-214 activated the AKT signaling pathway by directly targeting MIR31HG and PTEN. In addition, the tocilizumab-MIR31HG-miR-214-PTEN-AKT axis regulated the proliferation, migration, and production of inflammatory molecules and matrix metalloproteinases (MMPs) in RA-FLS. Furthermore, co-culture experiments showed that this axis could inhibit the inflammatory phenotype of macrophages and protect chondrocytes. In summary, our study shows that tocilizumab suppresses RA-FLS inflammation by regulating the MIR31HG-miR-214-PTEN-AKT pathway, and presents new insights on RA pathogenesis and potential targets for RA therapy.

## INTRODUCTION

Rheumatoid arthritis (RA) is a chronic autoimmune disease characterized by synovitis, autoantibody production, and erosion of cartilage that severely affect the joints [1]. Although significant progress has been achieved in developing effective RA therapies, challenges of resistance to certain RA drugs and their toxicity remain [2]. Thus, elucidating the pathogenesis of RA and the development of effective therapies remains a priority.

The joint synovium includes the lining and subliming layers, which contain a variety of cells including the fibroblast-like synoviocytes (FLS) [3], synovial macrophages [4], T cells [5] and B cells [6], with the FLS being the most abundant cell type. Normal synovial tissue is characterized by a thin lining consisting of several layers of cells. However, in RA, this lining becomes inflamed, hyperplasic, and invades both the cartilage and bone. RA-FLS exhibit aggressive tumor-like phenotypes that can further exacerbate joint damage by invading the extracellular matrix (ECM) [7]. The mechanisms underlying this hyperplasia and cartilage erosion are not fully understood, and thus, further research studies are warranted.

Tocilizumab is approved for use on its own or in combination with other medications to treat moderate to severe RA in adults in whom at least one other medication has failed [8]. However, the specific effects of tocilizumab on RA-FLS inflammation and the related mechanisms remain unknown. Several recent studies have demonstrated the essential role of non-coding RNAs (ncRNAs) in the establishment and development of RA [9]. Thus, this study was designed to explore this area further. We found that tocilizumab inhibited FLS-mediated inflammatory phenotypes by inducing MIR31HG expression in the RA-FLS. Moreover, MIR31HG inhibited the proliferation, migration, and expression of inflammatory cytokines and MMPs by modulating the downstream miR-214-PTEN-AKT pathway in these cells. These findings possibly explain the mechanism underlying the action of this therapy in RA-FLS and proposes novel therapeutic targets for RA.

## RESULTS

### Tocilizumab mitigates FLS-mediated inflammation in RA

**Figure 1:**
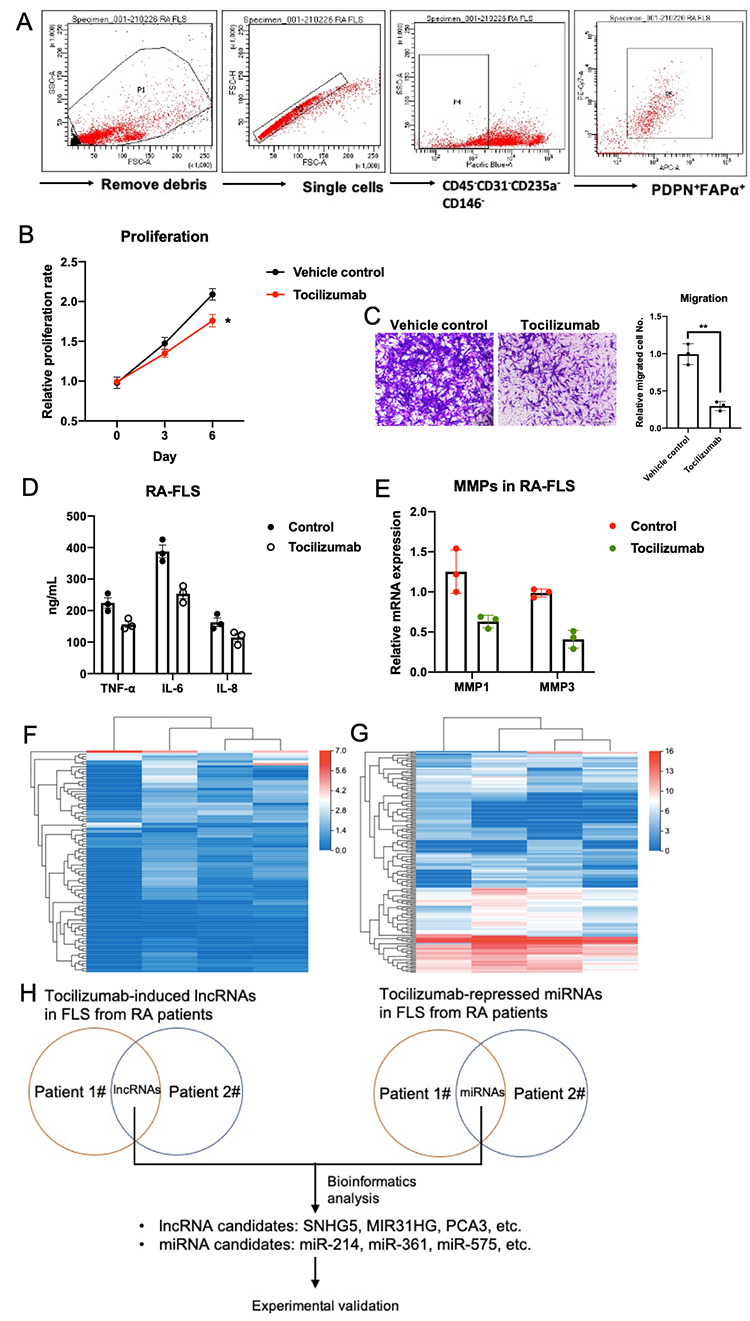
**Anti-inflammatory effects of tocilizumab on RA-FLS-mediated inflammation.** (**A**) The sorting strategy for isolating RA-FLS from joint synovium of RA patients. (**B**, **C**) The effects of tocilizumab on the proliferation and migration abilities of RA-FLS (n=3). (**D**, **E**) The effects of tocilizumab on various cytokines and MMPs of RA-FLS (n=3). (**F**, **G**) LncRNA-seq and miRNA-seq were performed using tocilizumab-treated RA-FLS. (**H**) Strategy for selecting potential tocilizumab lncRNA and miRNA targets in RA-FLS for downstream experimental validation. Data represent the mean ± SEM; *p < 0.05, as determined by the Student’s *t-*test.

We first isolated primary FLS from RA tissues and then cultured them *in vitro* to assess the effects of tocilizumab treatment on RA-FLS associated inflammation (Figure 1A). Tocilizumab inhibited the proliferation and migration of RA FLS (Figure 1B, 1C) and significantly inhibited the expression of various inflammatory cytokines and matrix metalloproteinases (MMPs) in these cells (Figure 1D, 1E). Our results suggest that tocilizumab treatment may reduce FLS-mediated inflammation in RA. Next, we aimed to identify the lncRNAs and miRNAs regulated by tocilizumab in these cells by performing lncRNA- and miRNA-seq (Figure 1F, 1G). After reviewing the data for each of the tocilizumab-induced lncRNA and tocilizumab-inhibited miRNA using bioinformatic analysis and available scientific literature, several lncRNAs (MIR31HG, SNHG5, PCA3) and miRNAs (miR-214, miR-361, miR-575) were selected for experimental validation (Figure 1H and Supplementary Figures 1, 2).

### MIR31HG, a lncRNA targeted by tocilizumab, suppresses RA-FLS inflammation

**Figure 2:**
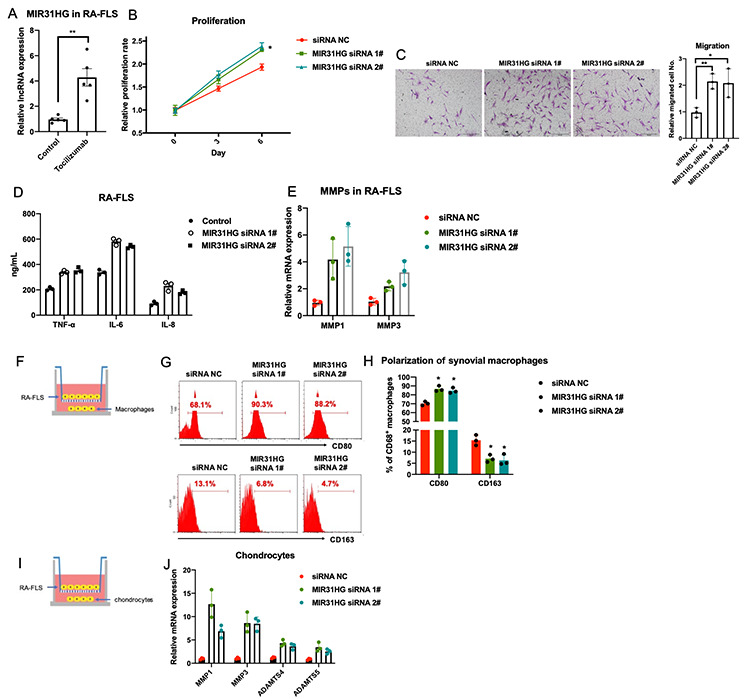
**The effects of lncRNA MIR31HG on RA-FLS-mediated inflammation.** (**A**) The effects of tocilizumab on endogenous expression of MIR31HG in RA-FLS (n=3). (**B**–**E**) MIR31HG knockdown affects proliferation, metastasis, and production of inflammatory molecules and MMPs in RA-FLS (n=3). (**F**–**J**) RA-FLS with reduced MIR31HG expression regulates primary macrophages and chondrocytes (n=3). Data represent the mean ± SEM; *p < 0.05, Student’s *t-*test.

Combining analysis of RNA-seq data, qPCR validation (Supplementary Figure 1A) and literature screening, the effects of MIR31HG, SNGH5 and PCA3 on RA-FLS proliferation were evaluated (Supplementary Figure 2) by CCK-8 assay. The results showed that only MIR31HG KD repress RA-FLS proliferation among these candidates. This analysis identified MIR31HG as a key tocilizumab-induced lncRNA and miR-214 as the key miRNA suppressed by tocilizumab in RA-FLS (Supplementary Figure 1B). Since MIR31HG is a validated target of miR-214 [10], we speculated that tocilizumab may regulate the MIR31HG-miR-214 axis in RA-FLS. Our qPCR results verified that tocilizumab treatment significantly increased the expression of MIR31HG in RA-FLS (Figure 2A). Knockdown of endogenous MIR31HG expression using specific siRNA (Supplementary Figure 3) increased the proliferation, metastasis, and production of inflammatory molecules and MMPs in RA-FLS (Figure 2B–2E). Furthermore, co-culture experiments showed that MIR31HG knockdown in RA-FLS indirectly promoted acquisition of inflammatory phenotypes by the macrophages and aggravated chondrocyte damage (Figure 2F–2J). Taken together, these data suggest that MIR31HG inhibits RA-FLS-mediated inflammation.

### The MIR31HG-miR-214-PTEN axis inhibits RA-FLS inflammation

**Figure 3:**
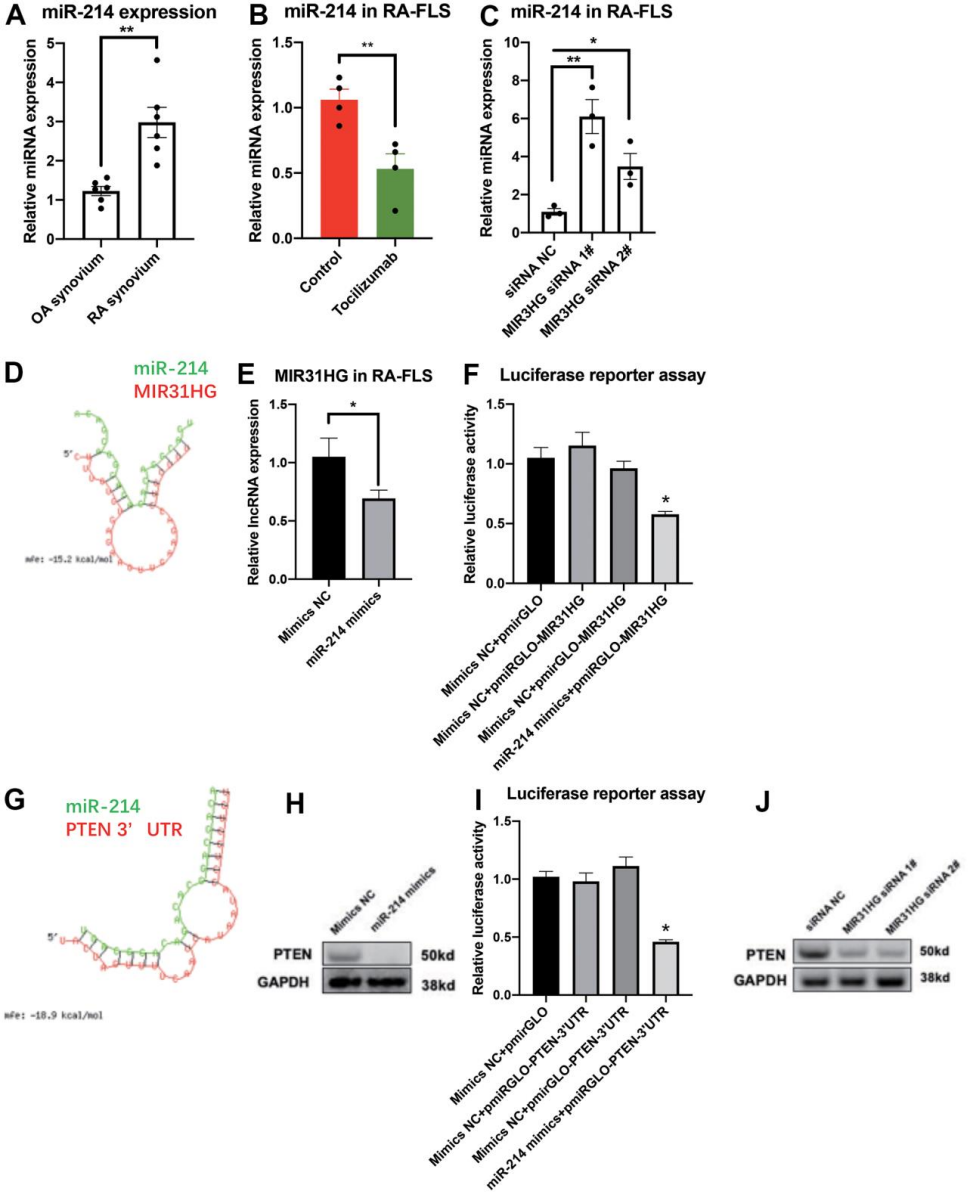
**The ceRNA network for MIR31HG-miR-214-PTEN in RA-FLS.** (**A**) The endogenous expression of miR-214 in OA and RA synovium samples (n=6). (**B**) The endogenous expression of miR-214 in RA-FLS following tocilizumab treatment (n=4). (**C**) The effects of MIR31HG siRNAs on miR-214 expression in RA-FLS (n=3). (**D**) Bioinformatic prediction of binding between MIR31HG and miR-214. (**E**) The expression of MIR31HG in miR-214 overexpressing RA-FLS (n=3). (**F**) Luciferase reporter assay demonstrating the direct interactions between miR-214 and MIR31HG (n=3). (**G**) Bioinformatic prediction of binding between the PTEN 3’ UTR and miR-214. (**H**) The expression of PTEN in miR-214 overexpressing RA-FLS (n=3). (**I**) Luciferase reporter assay demonstrating the direct interactions between miR-214 and the PTEN 3′ UTR (n=3). (**J**) Western blot showing the endogenous expression of PTEN in MIR31HG knockdown RA-FLS (n=3). Data represent the mean ± SEM; *p < 0.05, as determined by the Student’s *t-*test.

RNA-seq was used to study the function of miR-214 in RA-FLS. KEGG pathway analysis revealed that miR-214 overexpression closely associated with increase in the expression of several genes associated with multiple inflammatory signaling pathways in RA-FLS (Supplementary Figure 4). In addition, miR-214 expression was upregulated in RA synovium compared to the OA control (Figure 3A). Endogenous miR-214 expression was significantly downregulated in RA-FLS treated with tocilizumab (Figure 3B). In addition, siRNA mediated knockdown of MIR31HG led to the upregulation of miR-214 expression in RA-FLS (Figure 3C). The results of the bioinformatic analysis, qPCR, western blotting, and luciferase reporter assays demonstrated that miR-214 directly targeted MIR31HG (Figure 3D–3F), as well as PTEN, another anti-inflammatory target (Figure 3G–3I). In addition, negative correlations were observed between the expression of miR-214 and MIR31HG/PTEN in these cells (Supplementary Figure 5). Western blot analysis also confirmed that endogenous expression of PTEN was also inhibited in response to MIR31HG knockdown in RA-FLS (Figure 3J), suggesting that MIR31HG-miR-214-PTEN forms a regulatory network in RA-FLS.

**Figure 4:**
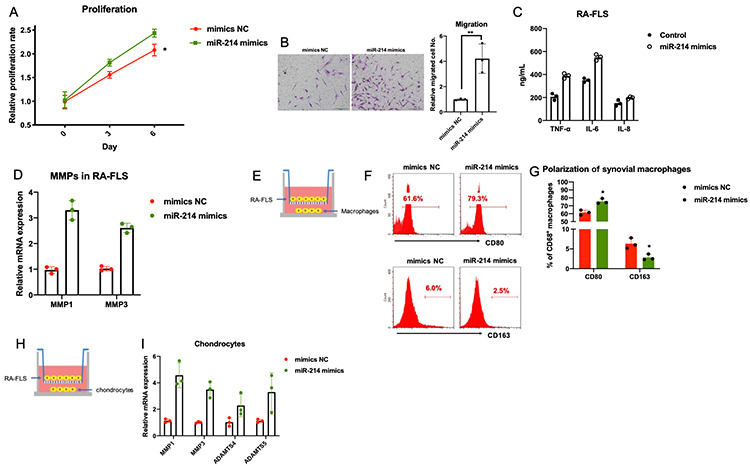
**The effects of miR-214 overexpression on RA-FLS-mediated inflammation.** (**A**–**D**) The effects of miR-214 overexpression on proliferation, metastasis, and production of inflammatory molecules and MMPs in RA-FLS (n=3). (**E**–**I**) The effects of miR-214 overexpressing RA-FLS on primary macrophages and chondrocytes (n=3). Data represent the mean ± SEM; *p < 0.05, Student’s *t-*test.

In addition, overexpression of miR-214 promoted the proliferation, migration, and expression of inflammatory molecules and MMPs in primary FLS obtained from patients with RA (Figure 4A–4D). Similar to the MIR31HG knockdown, co-culture experiments showed that miR-214 overexpression indirectly promoted inflammatory phenotypes in the macrophages and increased chondrocyte damage (Figure 4E–4I).

### The tocilizumab-MIR31HG axis suppresses the AKT pathway in RA-FLS

**Figure 5:**
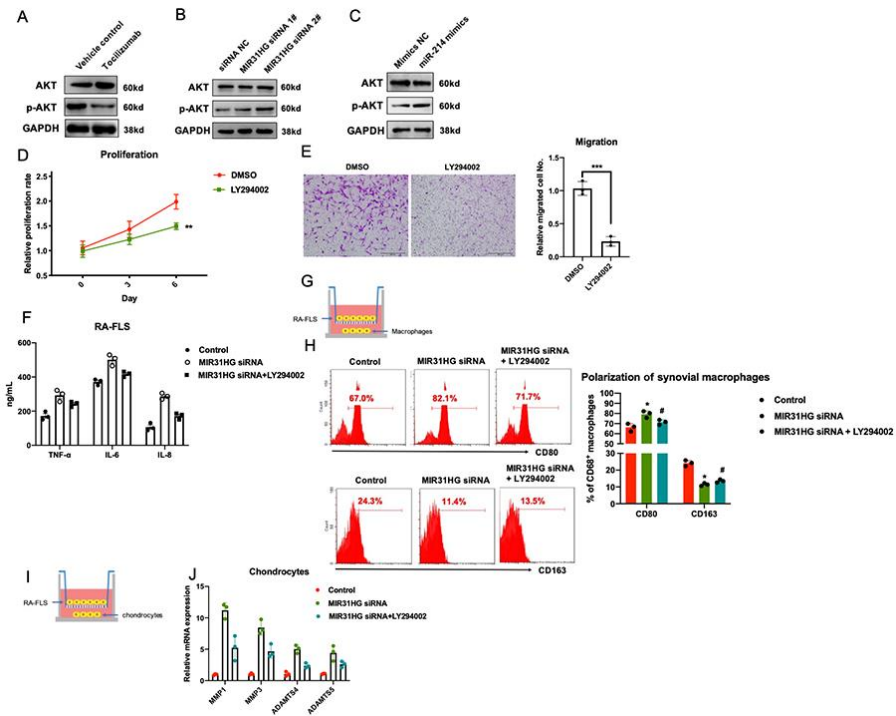
**The effects of the tocilizumab-MIR31HG axis on the AKT pathway in RA-FLS.** (**A**–**C**) The effects of tocilizumab, MIR31HG and miR-214 on the AKT pathway in RA-FLS. (**D**, **E**) The effects of AKT pathway inhibitor, LY294002 on the proliferation and migration of primary RA-FLS. (**F**) LY294002 mediated rescue of the MIR31HG knockdown-induced expression of inflammatory molecules in RA-FLS. (**G**–**J**) LY294002 mediated rescue of the MIR31HG knockdown-induced expression of inflammatory phenotypes in primary macrophages and chondrocytes using an *in vitro* co-culture system. Data represent the mean ± SEM; **p < 0.01, as determined by the Student’s *t-*test.

Based on the results from previous reports [11–13] and our RNA-seq results (Supplementary Figure 4), we identified the PI3K/AKT pathway as a potential downstream target of the tocilizumab-MIR31HG-miR-214-PTEN axis in RA-FLS. Western blot results showed that tocilizumab, MIR31HG, and miR-214 regulated p-AKT in RA-FLS (Figure 5A–5C). Next, we used an AKT inhibitor to confirm the function of the AKT pathway in RA-FLS. PI3K inhibitor LY294002 was used to block the AKT pathway in RA-FLS (Supplementary Figure 6) and it successfully inhibited the proliferation and migration of RA-FLS (Figure 5D, 5E). To validate that the AKT pathway is the downstream signaling pathway of MIR31HG, LY294002 was added to RA-FLS subjected to MIR31HG knockdown. The results of qPCR showed that the addition of LY294002 rescued MIR31HG knockdown-induced expression of inflammatory molecules in RA-FLS (Figure 5F) and restored the MIR31HG knockdown-induced inflammatory phenotype of primary macrophages and chondrocytes in the co-culture system (Figure 5G–5J). This suggests that the inhibitory effects of MIR31HG on RA-FLS mediated inflammation are at least partly mediated by suppressing the AKT pathway in these cells.

## DISCUSSION

Several studies have suggested that tocilizumab exerts anti-inflammatory effects by inhibiting the expression of both inflammatory chemokines and adhesion molecules, such as MCP-1, IL-8, and ICAM-1 [14]. In this study, we investigated the anti-inflammatory effects of tocilizumab in RA-FLS from the perspective of ncRNAs, including lncRNAs and miRNAs. Our results revealed that tocilizumab inhibited RA-FLS inflammation by increasing the expression of the lncRNA MIR31HG. In addition, silencing MIR31HG inhibited the proliferation, migration, and expression of inflammatory factors and MMPs in RA-FLS by inhibiting the miR-214-PTEN-AKT pathway. In addition, co-culture experiments with chondrocytes and macrophages showed that MIR31HG indirectly mitigated the damage to chondrocytes and the inflammatory response in macrophages. Thus, our results suggest that MIR31HG acts as a regulator of the anti-inflammatory response in RA-FLS, thereby suppressing increased cell proliferation, invasiveness, and inflammation associated with RA. Taken together, our data suggest that MIR31HG plays a protective role in RA-induced synovitis (Supplementary Figure 7).

The invasiveness of the FLS is one of the major causes of bone and cartilage damage in RA. To date, it is not clear how tocilizumab and MIR31HG modulate RA-FLS involved in joint degradation. Our findings shows that knocking down MIR31HG in primary RA-FLS promotes chondrocyte degradation. Similarly, MMP overexpression is one of the main factors responsible for pathological ECM reconstruction and cell migration in the joints of patients with RA. MIR31HG knockdown enhanced the expression of MMPs, including MMP1 and MMP3, suggesting that MIR31HG, is a positive downstream regulator of tocilizumab and may mitigate pathological features of RA by inhibiting the invasiveness of RA-FLS.

Competing endogenous RNAs (ceRNAs) are one of the most important regulatory agents for modulating lncRNA function. MIR31HG is a direct target of miR-214 in non-small cell lung cancer (NSCLC) [10], and miR-214 promotes inflammation and immunity in several systems, such as organ transplant and immune tolerance [15, 16]. In addition, PTEN is a direct target of miR-214 [17], and several studies have confirmed that PTEN plays an anti-inflammatory role in RA [12, 13, 18]. These findings combined with our bioinformatic, qPCR, western blot, and dual luciferase reporter assays, suggest that a ceRNA regulatory network comprising MIR31HG-miR-214-PTEN is involved in RA-FLS. These findings expands our understanding of the molecular mechanisms underlying RA-FLS-mediated inflammation. The AKT signaling pathways plays pro-inflammatory role in RA-FLS [19], but little has been reported on its upstream regulation. Here, we report that tocilizumab and MIR31HG inhibit the activation of the AKT pathway in RA-FLS, and that this effect is mediated, at least in part, via the MIR31HG-miR-214-PTEN ceRNA regulatory network. These data provide new insights on the anti-inflammatory mechanism of tocilizumab and suggest potential targets for RA therapeutics.

In the future, we intend to expand this investigation in several ways. First, since tocilizumab is an IL-6R antagonist, we would like to study the relationship between IL-6 and MIR31HG, and identify the direct regulator of MIR31HG in the IL-6 signaling pathway. Our data showed that MIR31HG knockdown significantly increased IL-6 production (Figure 2D) and MIR31HG expression was induced in RA-FLS subjected to IL-6 knockdown (Supplementary Figure 8), suggesting the existence of a negative feedback loop between these two effectors. Furthermore, the number of RA patients included in this study was relatively small; therefore, we will continue to recruit more RA patients and healthy donors to validate our results in a larger clinical cohort. Finally, we plan to use murine arthritis models to further assess the effects of the tocilizumab-MIR31HG-miR-214-PTEN-AKT pathway on RA inflammation *in vivo*.

## MATERIALS AND METHODS

### Patients

The synovial tissue samples were obtained from patients with RA (all Chinese) who underwent synovectomy at the First Affiliated Hospital of Anhui Medical University. The current experiments were approved by the Ethics Committee of the First Affiliated Hospital of Anhui Medical University. The diagnosis of OA (six female patients, average age=54.5 years, age range: 48~63 years) and RA (one male and five female patients, average age=56.67 years, age range: 50~64 years) conformed to the criteria of the American College of Rheumatology (ACR) [20]. Trauma, sepsis, or tuberculosis-induced OA and RA were excluded from this study and none of the patients included in this study took any medications before their corrective surgery.

### Isolation and *in vitro* culture of FLS, macrophages and chondrocytes from patients with RA

Macrophage, chondrocyte, and FLS isolation from RA synovial tissues was performed as previously described [21]. Briefly, the synovial membrane was excised from the OA and RA patients, and minced in Dulbecco’s modified Eagle medium (DMEM; Gibco) containing 10% fetal bovine serum (FBS; Gibco) and streptomycin/penicillin (Gibco). The synovial tissue was then digested in DMEM medium containing collagenase type IV (1 mg/mL; Sigma Aldrich) and 0.1 mg/mL deoxyribonuclease I (Sigma Aldrich), and incubated at 37° C for 2 h. The digested tissues were vortexed and resuspended in DMEM medium before centrifugation at 3,000 rpm for 5 min before being resuspended in PBS and subjected to cell sorting. Sorted For RA synovium chondrocytes, the cartilage from RA patients was minced and then treated with collagenase II (Sigma) with agitation (200 rpm) at 37 ^o^C for 8 h. The cell suspension was filtered by using a cell strainer (40 μm), washed with PBS, centrifuged at 3,000 rpm for 10 min, resuspended in 10 mL DMEM supplemented with 10% FBS and penicillin/streptomycin, and then routinely cultured in a humidified incubator (37° C and 5% CO_2_).

### Flow cytometry analysis of primary FLS and macrophages

Isolated FLS were incubated with the antibodies specific to the following proteins at 4° C for 1 h to facilitate sorting: CD45-FITC(130-110-769, Miltenyi), CD31-FITC(130-117-312, Miltenyi), CD146-FITC(130-097-934, Miltenyi), CD235a-FITC(130-117-800, Miltenyi), hFAPα-APC(FAB3715A-025, R&D Systems), PDPN-PE(130-117-799, Miltenyi), CD80-APC(130-117-719, Miltenyi), and CD163-APC(130-112-129, Milteny). Myeloid cells identified by CD45 expression, endothelial cells by CD31 expression, red blood cells by CD235a expression, and pericytes by CD146 were excluded. Two FLS markers (PDPN and FARα) were applied to identify FLS. For primary macrophage sorting, single cells from the RA synovium samples were centrifuged, the cell pellet was resuspended in fresh RPMI 1640 media, and sorted to obtain cells via CD45-FITC^+^CD15-APC^−^CD1c-APC^−^CD14-PE^+^ strategy using FACSAria II cell sorter (BD Biosciences). Unlabeled cells were used as the negative control in the other flow cytometry experiments described in this paper. The sorted FLS were cultured in DMEM supplemented with 10% FBS and penicillin/streptomycin at 37° C and 5% CO_2_ and the culture medium was replenished every two days, until cells reached 90% confluence. The other experiments from this study were completed using primary RA-FLS from passages 2 to 6. Primary macrophages were cultured in Macrophage Base Medium XF (PromoCell) for 7–10 days prior to their use in any experiment.

### RA-FLS transfection

RA-FLS were transfected with miR-214 mimics (100 nM, sense sequence: 5′-ACAGCAGGCACAGACAGGCAGU-3′, anti-sense sequence: 5′- UGCCUGUCUGUGCCUGCUGUUU-3′) and the scrambled control (GenePharma) by using Lipofectamine RNAiMAX (Invitrogen). Cells were lysed 48-72 h after transfection and the lysates were used in the downstream experiments. Small interfering RNAs (siRNAs) against MIR31HG and IL-6 were purchased from GenePharma. The cell stimulation experiments were performed using RA-FLS treated with tocilizumab (0.5 ng/mL) and AKT pathway blocker, LY294002(20 μM/mL).

### CCK-8 assay

Cell proliferation was evaluated using the CCK-8 kit (DOJINDO) as per the manufacturer’s protocol. Briefly, in a 96-well plate, cell suspensions were added to each well (5000 cells in 100 μL/well) followed by the addition of 10 μL CCK-8 reagent. Plates were incubated for 1 h before the absorbance was measured at 450 nm using a microplate reader.

### Cell migration assay

Migration of FLS was evaluated using transwell assay (Corning), after they were treated with tocilizumab, MIR31HG siRNA, miR-214 mimics, and LY294002. The cells (5,000 cells/well) were seeded in the upper chamber of the transwell plate and incubated for 12 h in a humidified CO_2_ incubator at 37° C. We evaluated the migration of the treated RA-FLSs by measuring their migration through the membrane of the upper chamber and the RA-FLSs attached to the lower membrane were stained with 0.1% crystal violet and visualized under a light microscope. The average number of migrated cells were estimated by counting the number of cells in six random fields of view.

### Real-time PCR

RNA was extracted by using TRIzol (Invitrogen). The extracted RNA was washed and resuspended in nuclease-free water before being treated with DNase I. Total RNA was quantified and its purity was evaluated using a Nanodrop 2000 (Thermo Fisher Scientific). The mRNA reverse transcription kit (Takara) and TaqMan reverse transcription kit (Life Technologies) were used for reverse-transcribing mRNA and miRNA, respectively, as per manufacturer’s instructions.

Universal SYBR Green Master mix (Applied Biosystems) was used to perform quantitative PCR (qPCR) for analyzing the mRNA expression, while Hairpin-itTM microRNA qPCR Quantitation Kit (GenePharma) was applied to detect miR-214 in RA-FLS. All qPCR experiments were completed on a 7500 real-time PCR system (Applied Biosystems); GAPDH and U6 small nucleolar RNA (snoRNA) were used as endogenous controls for normalizing the expression of the mRNA and miRNAs, respectively.

### ELISA

Production of inflammatory cytokines by the RA-FLS was evaluated using commercial ELISA kits. The supernatants were collected from each group and evaluated for TNF-α, IL-6, and IL-8 expression using respective ELISA kits (R&D Systems) according to the manufacturer’s instructions.

### Western blot

RA-FLSs were lysed using ice-cold lysis buffer, and total proteins were extracted and quantified using a protein quantification assay. Equal volumes of protein were mixed with loading buffer and resolved by performing SDS-PAGE. Resolved proteins were transferred to polyvinylidene fluoride membranes and then incubated with the following primary antibodies: anti-PTEN antibody (Abcam, 1:1000), anti-AKT antibody (Cell Signaling, 1:2000), and anti-p-AKT antibody (Proteintech, 1:2000). The membranes were then washed and incubated with horseradish peroxidase-conjugated secondary antibodies and the binding signal was visualized using an enhanced chemiluminescence system (GE Systems).

### RNA- and microRNA-seq

Total RNA was extracted from the FLS following treatment with tocilizumab or transfection with MIR31HG siRNA or miR-241 mimics. The RNA quality and quantity was evaluated using a NanoDrop200 before being used to prepare the libraries for the RNA-seq. For the miRNA-seq, total RNA was purified on a 15% urea denaturing gel, and small RNA regions, corresponding to the 18–30 nucleotide, were excised and recovered. These small RNAs were ligated to adenylated 3′ adapters annealed to unique molecular identifiers (UMI) followed by annealing to 5′ adapters. These adapter-ligated small RNAs were transcribed into cDNA using SuperScript II Reverse Transcriptase (Invitrogen), and then PCR amplified using specific primer cocktail and PCR mix to enrich the specific miRNA cDNA fragments. The PCR products were evaluated by agarose gel electrophoresis and those corresponding to 110–130 bp were purified using the QIAquick Gel Extraction Kit (QIAGEN). This library was (1) checked for the distribution of the fragment size using the Agilent 2100 bioanalyzer, and (2) quantified using qPCR (TaqMan Probe). The final PCR products were sequenced using the BGISEQ-500 platform (BGI).

### Luciferase reporter assay

We cloned ~500 bp of the MIR31HG and human PTEN 3’-untranslated region (3’ UTR), which included the miR-214 seed sequence, into pmirGLO reporter vector (Promega) to produce luciferase reporter plasmids. HEK293T cells were plated in 12-well plates and transfected with 1 mg of each reporter plasmid, 20 nM of the miRNA mimics, or scrambled controls (NC). Luciferase activity was measured 48 h after transfection using the Dual-Luciferase Reporter Assay System (Promega) and GloMax 20/20 LUMINOMETER (Promega).

### Statistical analysis

Normality of the data was checked and the statistical analysis was completed using SPSS software (version 13.0). As all samples were normally distributed, subsequent comparisons were completed using the Student’s *t*-test, while the correlation analysis was performed using Pearson’s analysis. Data was considered significant at *p <0.05, **p <0.01 and ***p <0.001.

## Supplementary Material

Supplementary Figures
