# Supplementary material for: LncRNA MIR31HG is induced by tocilizumab and ameliorates rheumatoid arthritis fibroblast-like synoviocyte-mediated inflammation via miR-214-PTEN-AKT signaling pathway
Source: Aging (Albany NY). 2021 Nov 9;13(21):24071–85. doi: 10.18632/aging.203644 (PMC8610144; doi:10.18632/aging.203644)
Supplement: Supplementary Figures [file aging-13-203644-s001.pdf]

SUPPLEMENTARY FIGURES

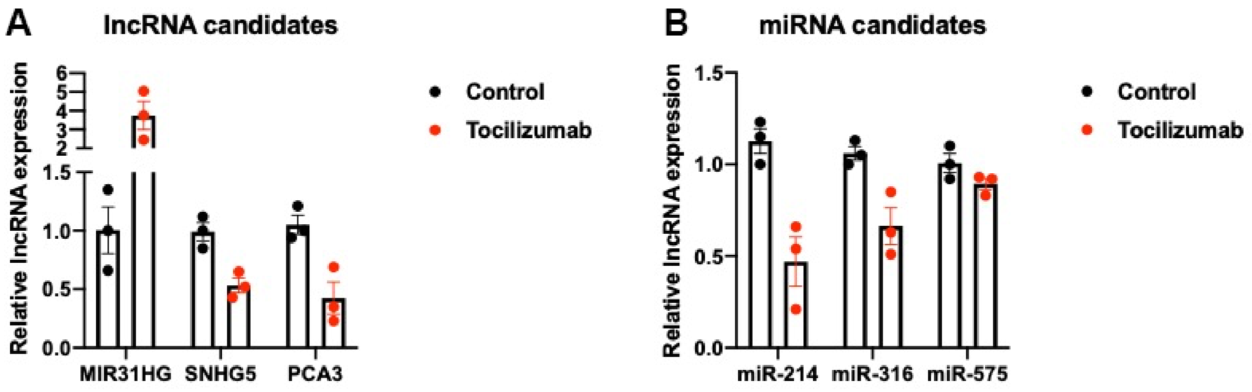

Supplementary Figure 1. The candidates of (**A**) lncRNAs (MIR31HG, SNHG5, PCA3) and (**B**) miRNAs (miR-214, miR-361, miR-575) were selected for experimental validation.

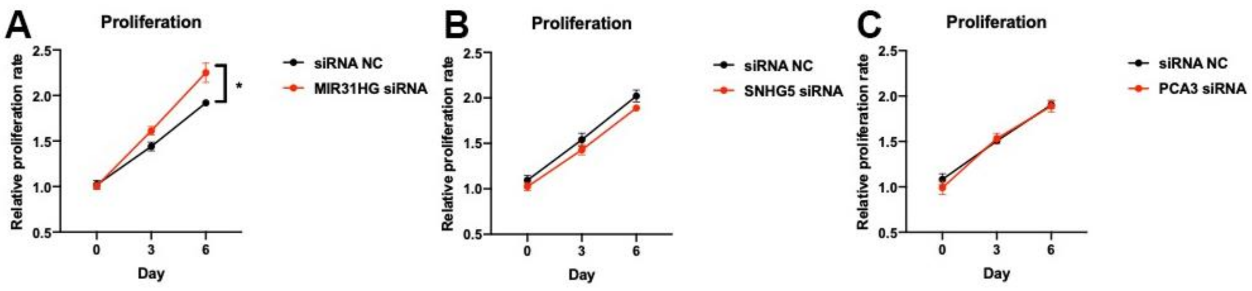

Supplementary Figure 2. The effects of knockdown of lncRNAs candidates (**A**) MIR31HG, (**B**) SNHG5 and (**C**) PCA3 on proliferation of RA-FLS. Data represent means ± SEM; *p < 0.05, as determined by the Student's t test.

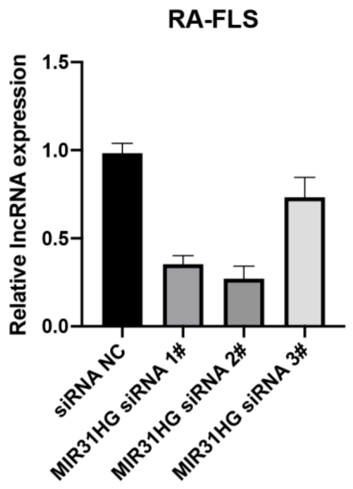

Supplementary Figure 3. The knockdown efficiency of MIR31HG siRNAs in RA-FLS.

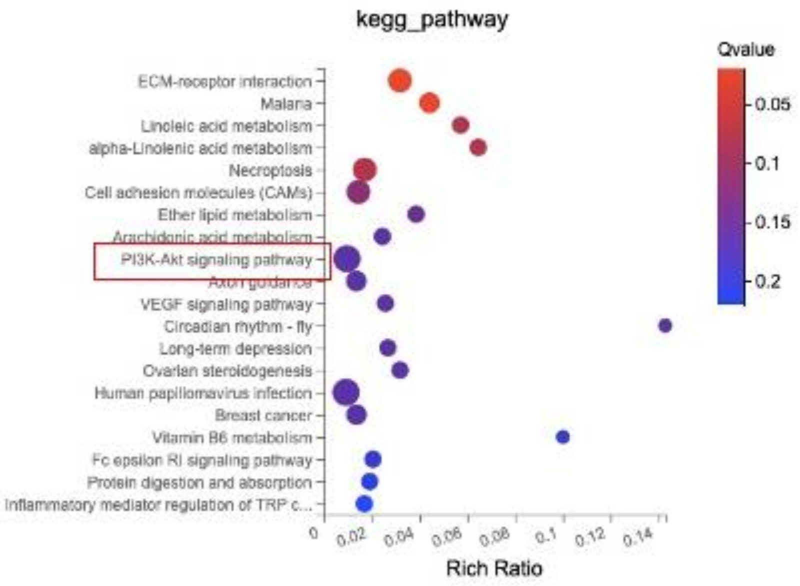

Supplementary Figure 4. The KEGG pathway analysis of miR-214 overexpression RA-FLS.

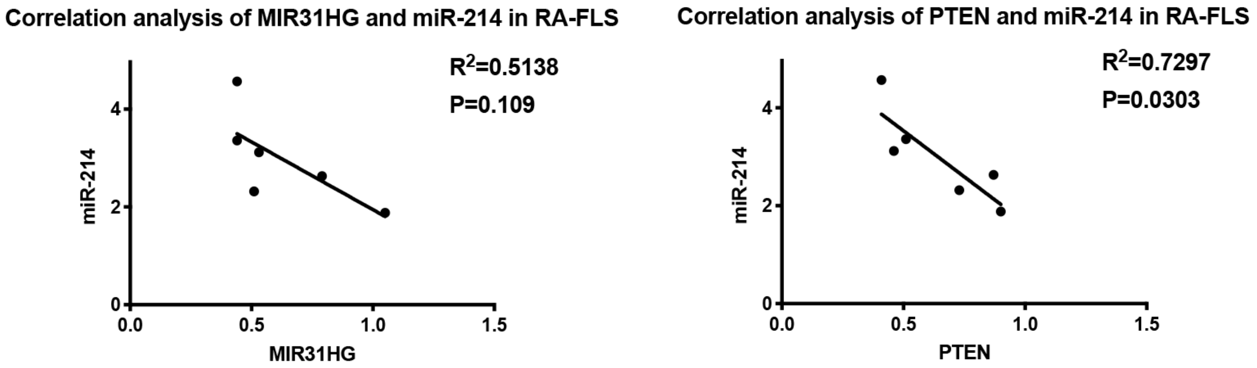

Supplementary Figure 5. The correlation coefficient between MIR31HG and miR-214 or PTEN in RA-FLS.

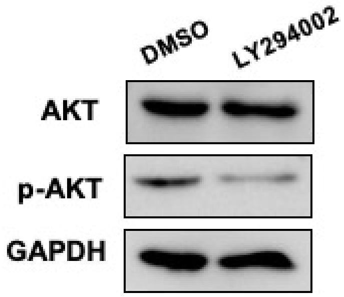

Supplementary Figure 6. The validation of the inhibitory effect of LY294002 on AKT pathway in RA-FLS.

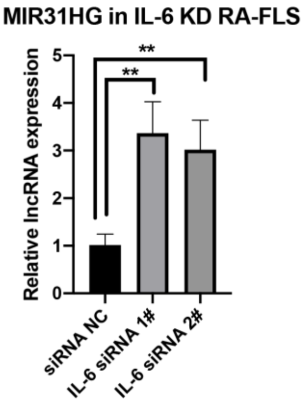

Supplementary Figure 7. The expression of MIR31HG in IL-6 KD RA-FLS.

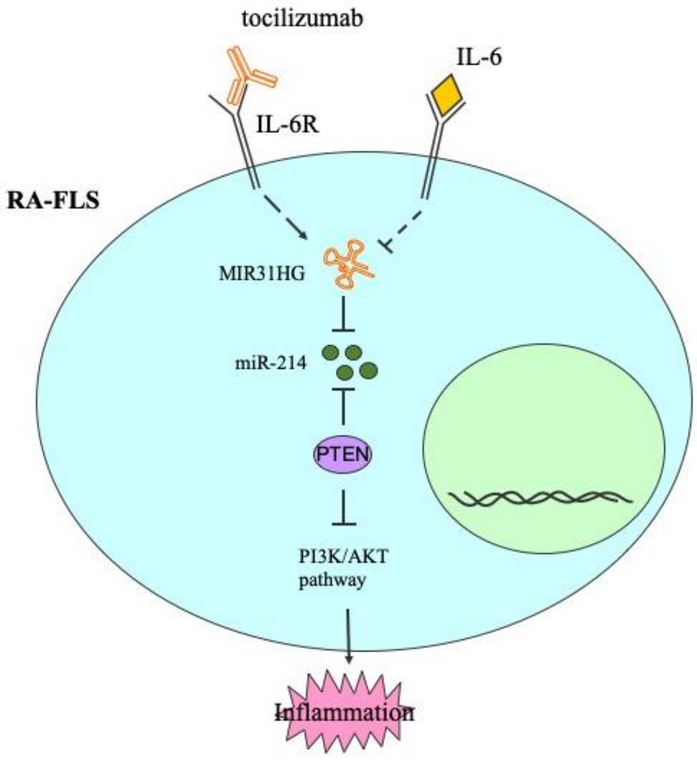

Supplementary Figure 8. The graphic abstract of the current manuscript.

Gene ID	Gene Symbol	Type	log2 (B3 / B1)	FDR (B3 / B1)
BGIG9606_55819	'BGIG9606_55819'	lncRNA	7.01122726	4.54E-08
554202	'MIR31HG'	lncRNA	5.70043972	3.91E-04
100533184	'ARHGAP19-SLIT1'	lncRNA	5.52356196	1.96E-05
BGIG9606_46824	'BGIG9606_46824'	lncRNA	4.95419631	6.51E-05
BGIG9606_58794	'BGIG9606_58794'	lncRNA	4.42626475	1.05E-04
BGIG9606_51809	'BGIG9606_51809'	lncRNA	4.169925	3.37E-08
BGIG9606_44551	'BGIG9606_44551'	lncRNA	4.02974734	9.59E-04
105378345	'LOC105378345'	lncRNA	4	3.22E-04
BGIG9606_59237	'BGIG9606_59237'	lncRNA	3.84130225	4.48E-06
105376244	'LOC105376244'	lncRNA	3.80735492	9.28E-04
107987330	'FAM239C'	lncRNA	3.70043972	9.59E-04
BGIG9606_50097	'BGIG9606_50097'	lncRNA	3.5849625	8.36E-08
399717	'GATA3-AS1'	lncRNA	3.32192809	1.84E-04
100534612	'C1QTNF3-AMACR'	lncRNA	3.169925	2.58E-06
BGIG9606_45073	'BGIG9606_45073'	lncRNA	3.02974734	6.32E-05
BGIG9606_47247	'BGIG9606_47247'	lncRNA	3	2.12E-09
105376373	'LOC105376373'	lncRNA	2.63742992	2.36E-04
BGIG9606_40631	'BGIG9606_40631'	lncRNA	2.5849625	7.08E-04
BGIG9606_50404	'BGIG9606_50404'	lncRNA	2.50250034	6.09E-04

Gene ID	Gene Symbol	Type	log2 (B3 / B1)	FDR (B3 / B1)
hsa-miR-9-5p		miRNA	-13.621136	7.87E-44
hsa-miR-138-2-3p		miRNA	-12.036174	1.42E-14
hsa-miR-316-5p		miRNA	-12.036174	1.43E-14
hsa-miR-135a-5p		miRNA	-11.929258	6.78E-14
hsa-miR-575-5p		miRNA	-11.550747	1.65E-10
hsa-miR-664b-3p		miRNA	-11.287712	8.12E-09
hsa-miR-214-5p		miRNA	-11.228819	1.76E-08
hsa-miR-1225-5p		miRNA	-11.167418	3.82E-08
hsa-miR-106b-5p		miRNA	-11.103288	1.77E-08
hsa-miR-2278		miRNA	-11.036174	1.76E-07
hsa-miR-6770-5p		miRNA	-10.965784	3.73E-07
hsa-miR-365a-5p		miRNA	-10.891784	8.17E-07
hsa-miR-4750-5p		miRNA	-10.813781	1.78E-06
hsa-miR-4647		miRNA	-10.813781	1.77E-06
hsa-miR-142-5p		miRNA	-10.550747	1.78E-05
hsa-miR-4440		miRNA	-10.550747	1.77E-05
hsa-miR-219a-5p		miRNA	-10.550747	1.76E-05
hsa-miR-302d-3p		miRNA	-10.451211	3.78E-05
hsa-miR-216a-5p		miRNA	-10.451211	3.80E-05

Supplementary Figure 9. Top 20 up-regulated lncRNAs and down-regulated miRNAs in tocilizumab-treated RA-FLS.
